# Mapping developmental transitions in mental health from mid‐ to late‐adolescence: Concurrent and longitudinal links to cognition

**DOI:** 10.1002/jcv2.70016

**Published:** 2025-06-05

**Authors:** Silvana Mareva, Jenna Parker, Marc Bennett, Laura Pass, Tobias Banaschewski, Arun L. W. Bokde, Sylvane Desrivières, Herta Flor, Sarah Hohmann, Dimitri Papadopoulos Orfanos, Hugh Garavan, Penny Gowland, Henrik Walter, Rüdiger Brühl, Jean‐Luc Martinot, Marie‐Laure Paillère Martinot, Eric Artiges, Frauke Nees, Tomáš Paus, Luise Poustka, Michael N. Smolka, Nilakshi Vaidya, Robert Whelan, Gunter Schumann, Joni Holmes

**Affiliations:** ^1^ Psychology Department Faculty of Health and Life Sciences University of Exeter Exeter UK; ^2^ MRC Cognition and Brain Sciences Unit University of Cambridge Cambridge UK; ^3^ Department of Clinical Psychology University of East Anglia Norwich UK; ^4^ Department of Child and Adolescent Psychiatry and Psychotherapy Central Institute of Mental Health Medical Faculty Mannheim Heidelberg University Mannheim Germany; ^5^ School of Medicine Trinity College Institute of Neuroscience Trinity College Dublin Dublin Ireland; ^6^ Social, Genetic and Developmental Psychiatry Centre Institute of Psychiatry, Psychology and Neuroscience King's College London London UK; ^7^ Department of Psychology University of Mannheim Mannheim Germany; ^8^ Departments of Psychiatry and Psychology University of Vermont Burlington Vermont USA; ^9^ Sir Peter Mansfield Imaging Centre School of Physics and Astronomy University of Nottingham Nottingham UK; ^10^ Department of Psychiatry and Psychotherapy CCM Berlin Institute of Health Humboldt‐Universität zu Berlin Berlin Germany; ^11^ Physikalisch‐Technische Bundesanstalt (PTB) Braunschweig Germany; ^12^ Institut National de la Santé et de la Recherche Médicale Ecole Normale Supérieure Centre Borelli Universite Paris‐Saclay Paris France; ^13^ Department of Child and Adolescent Psychiatry Sorbonne Université Paris France; ^14^ Department of Psychiatry University of Montreal Montreal Quebec Canada; ^15^ Department of Child and Adolescent Psychiatry Center for Psychosocial Medicine University Hospital Heidelberg Heidelberg Germany; ^16^ Department of Psychiatry and Neuroimaging Center Technische Universität Dresden Dresden Germany; ^17^ Department of Psychiatry and Neuroscience Centre for Population Neuroscience and Stratified Medicine (PONS) Charité Universitätsmedizin Berlin Berlin Germany; ^18^ School of Psychology and Global Brain Health Institute Trinity College Dublin Dublin Ireland; ^19^ School of Psychology University of East Anglia Norwich UK

**Keywords:** adolescence, cognition, mental health, transitions

## Abstract

**Background:**

Developmental changes in mental health are mostly mapped between childhood and adolescence or childhood and adulthood. This study maps developmental transitions in mental health profiles from mid‐ to late‐adolescence, exploring how these transitions relate to cognitive function in mid‐adolescence.

**Method:**

Participants from the IMAGEN cohort (*N* = 1304) were followed from mid‐ (14 years) to late (22 years) adolescence. K‐means clustering was applied to data from those with elevated mental health problems to identify common profiles of mental health symptoms at each timepoint (*n* = 784 at 14 years, *n* = 655 at 22 years). Those with no mental health symptoms formed a comparison group (*n* = 520 at 14 years, *n* = 649 at 22 years). Transitions between the groups were mapped across time and related to cognitive function at age 14.

**Results:**

Three distinct mental health profiles were identified: presentations of externalising, internalising, or social problems. These were similar in mid‐ and late adolescence. Externalising problems were more common in mid‐adolescence. Persistent externalising and social problems were related to cognitive function in mid‐adolescence, but problems that emerged or resolved in late adolescence were not.

**Conclusions:**

These data highlight the importance of understanding the developmental context in which mental health symptoms occur, and the cognitive factors linked to their persistence.

**Key Points**

- Current approaches to understanding the developmental course of mental health difficulties rarely consider changes in profiles into late adolescence, meaning we know little about how mental health presentations might alter as adolescents prepare for adulthood.
- Mental health profiles were mapped in a large sample at two time points: at age 14, and again at age 22.
- Three profiles of mental health difficulties were identified at each time point. These were broadly similar, capturing either relatively more severe externalising symptoms, relatively more severe internalising problems, or greater difficulties with social relationships.
- For some people, their profiles changed over time (e.g., a more externalising profile at age 14 became more internalising by age 22). For other people, their difficulties persisted and did not change.
- Difficulty adjusting risk‐related behaviour was linked to persistent externalising and social problems from mid‐ to late‐ adolescence, and poor spatial working memory was linked to persistent externalising problems.
- These findings illustrate the need to recognise shifts in adolescent mental health, and the cognitive factors that confer risk, to move towards proactive models of mental health support

## INTRODUCTION

Adolescence is a period of heightened biological, cognitive, and social change associated with increased risks for mental health difficulties (Blakemore & Mills, 2014). Worldwide, the prevalence of mental health disorders increases from childhood to adolescence, peaking during late adolescence as young people develop a sense of identity and transition into adulthood (Beauchamp et al., 2018). Current approaches to understanding developmental changes in mental health focus on mental health profiles or diagnostic status at a single timepoint or developmental stage, and few explore transitions in symptom presentation into *late* adolescence (Bartels et al., 2018; Basten et al., 2016; Bathelt et al., 2021; Benhamou et al., 2023; Copeland et al., 2014; Vaillancourt et al., 2013). This period, spanning the late teenage years into the mid‐twenties (Patton et al., 2018), is associated with changes in living circumstances, increased vulnerability to substance abuse, and reduced access to mental health services (Blanco et al., 2008; Kessler et al., 2005). To move towards proactive models of intervention we need to understand how symptoms change in late adolescence, and what predicts these changes. In the current study, we map changes in mental health symptoms from mid‐ to late adolescence and explore their associations with cognition.

Developmental trajectories of mental ill‐health are typically mapped according to the prevalence of diagnostic disorders at different ages (Copeland et al., 2014). Diagnostic approaches run counter to a wealth of evidence showing that mental health disorders are highly comorbid, heterogeneous, and variable across the lifespan (Dalgleish et al., 2020). We therefore adopt a transdiagnostic approach that promotes the use of data‐driven approaches to delineate more refined phenotypes along dimensions of interest (Astle et al., 2021). Our method of choice was cluster analysis, which allows for the identification of data‐driven subgroups with similar phenotypes that crosscut diagnostic boundaries and align better with underlying neurobiology than diagnostic groupings (Mareva et al., 2023; Vaidya et al., 2020). Few studies have used this approach to track the developmental course of mental health. Exceptions are Basten et al. (2016) who adopted a similar approach using latent profile analysis to explore transitions in internalising and externalising behaviours in preschoolers (Basten et al., 2016), Bathelt et al. (2021) who tracked profiles of mental health difficulties from childhood (10 years) to adolescence (16 years), and Benhamou et al. (2025) who identified profiles across similar measures at 5, 11 and 17 years. Bathelt et al. (2021) showed that while externalising symptoms were common in childhood, internalising symptoms were more prominent in adolescence. Similarly, Benhamou et al. (2025) showed that fewer profiles of difficulties profiles were present at younger ages, with a rise in the number of different profiles and their complexity during middle childhood, followed by a reduction in the number of distinct profiles as participants approached late adolescence. In both studies, better family resources predicted changes in the presentation of mental health problems across development. Bathelt et al. (2021) additionally showed that better cognitive performance in childhood was associated with a reduction in behavioural problems in adolescence.

### The current study

A data‐driven clustering approach was used to identify mental health profiles in a sample of 1304 participants from the IMAGEN cohort (Schumann et al., 2010) across mid‐ to late adolescence (14–22 years), an understudied period associated with heightened risk for mental health problems (Beauchamp et al., 2018). Clusters of mental health problems were identified at each timepoint using self‐reports on the Strengths & Difficulties Questionnaire (SDQ) (Goodman, 1997). Transitions across these clusters were tracked over developmental time. Associations between these changes and cognitive function were explored because late adolescence is marked by changes in social roles and responsibilities, and adjustments to personal goals and motivations, that require effortful cognitive control to attenuate and regulate behaviour and emotions (Crone & Dahl, 2012). Measures of delay aversion and risk‐taking were included because impulsive/risky decision‐making increases in adolescence (Rosenbaum & Hartley, 2019) and because problems both inhibiting impulsive responses and delaying gratification are associated with elevated externalising symptoms (Nigg et al., 2006). A measure of ‘cold’ cognition with no affective component was included to explore whether cognitive function in the absence of emotional content predicted changes in mental health.

We had no hypotheses about the mental health profiles that would emerge, the concurrent cognitive features differentiating them, their developmental course, or the cognitive factors that would predict these changes. Instead, this was designed as a data‐driven exploratory study addressing four broad questions: (1) can we identify robust subgroups of adolescents presenting with distinct profiles of mental health at mid‐ and late‐ adolescence within a large community sample; (2) do the subgroups differ in terms of cognitive function; (3) how do individuals transition across clusters over developmental time, and; (4) does baseline cognitive function differ between those with persisting, resolving or emerging mental health problems from mid‐ to late adolescence?

## MATERIALS AND METHODS

### Participants

Participants were drawn from the IMAGEN cohort (Schumann et al., 2010) (*N* = 1304, T1: *M*age = 13.95 years, SD = 0.45; T2: *M*age = 22.1 years, SD = 0.71, 53% female). Those aged 13–15 at the cognitive baseline assessment, with mental health data at both timepoints, were included.

### Materials and procedure

Data were drawn from the IMAGEN consortium, a longitudinal study exploring the biological, psychological, and environmental factors that influence brain development and mental health during adolescence. Data were collected from eight sites across four European countries: London and Nottingham in the UK, Berlin, Hamburg, Mannheim and Dresden in Germany, Paris in France, and Dublin in Ireland. Ethical approval was obtained from the local research ethics committee at each site, and written consent from parents/carers or the participants, or verbal assent from the participants, was obtained prior to testing. Participants received compensation of up to £100 in either bank transfers or vouchers, depending on recruitment site and participation in the follow‐up assessments. Data were collected when participants were aged approximately 14, 16, 19 and 22 (Schumann et al., 2010). Here, we analyse mental health and cognitive data from the first (when participants were aged ∼14 years, referred to as baseline/T1 from hereon) and the final assessment points (when participants were ∼22 years old, referred to hereafter as T2/follow‐up). The data analysed include self‐report measures of mental health and performance on two cognitive tasks that were administered at both time points.

#### Mental health

Self‐report versions of the SDQ (Goodman, 1997) were administered at T1 and T2. Age‐uncorrected subscale scores (Emotional Problems, Hyperactivity, Conduct Problems, Peer Problems, Prosocial Behaviour) were used in all analyses to allow for easier comparisons with other studies using the SDQ. Higher scores indicated more severe difficulties for all scales except Prosocial where high scores indicate fewer difficulties.

#### Cognition

Two measures from the Cambridge Neuropsychological Test Automated Battery (Robbins & Sahakian, 2003) were administered at both timepoints (see Supporting Information S1 for task descriptions and scoring).

##### Cambridge gambling task (CGT)

The Cambridge gambling task (CGT) was used to measure risk‐tasking and delay aversion. Measures of delay aversion/impulsivity, risk‐taking, and risk adjustment were used as they reflect cognitive functions linked to risky behaviour and mental health problems in adolescence (Nigg et al., 2006). For the impulsivity/delay aversion score, larger negative values (lower values) corresponded to impulsive betting. For risk‐taking, higher values corresponded to being better able to tolerate risks (e.g., less risk‐averse). For risk adjustment, lower scores represented being poorer at adjusting risk behaviour.

##### Spatial working memory (SWM)

A self‐ordered, serial search task was used to measure SWM. A higher score corresponded to a greater number of errors, indicating poorer working memory.

### Analysis plan

A k‐means clustering algorithm was applied across the SDQ subscales separately for T1 and T2 for participants who showed elevated mental health problems on at least one of the SDQ subscales. The robustness of the solution was explored using 10‐fold cross‐validation calculating the mean silhouette score across folds. This approach was also used by Benhamou et al. (2025) in a study tracking transitions of mental health difficulties from childhood to adolescence in a different sample. We choose to implement it here because it ensures that the clustering space is not dominated by participants with scores in the typical range and is therefore optimised to capture variance, and therefore different clusters, among those with difficulties. Additionally, it allows us to directly compare our results to those reported in their independent sample. SDQ ratings and cognitive performance were then compared between each of the derived subgroups and those who did not have elevated mental health problems at T1 and T2, who formed a comparison group. The number of participants who moved across the derived clusters from T1 to T2 was then calculated, and the cognitive function of individuals with persisting, emerging, or resolving mental health problems was explored. The details for each step of the analysis are presented below. All analyses were completed in R‐version 4.2.0 (for individual packages see Supporting Information S1).

#### Data screening

Baseline (T1) SDQ profiles were compared between participants with and without SDQ data at T2 (Table S2). Those without follow‐up data had significantly elevated conduct problems and hyperactivity/inattention, and significantly lower levels of prosocial behaviour at T1, but the effect sizes were small (*p* < .05, *d* = −0.24, −0.21, and 0.15 respectively). The analysis reported below includes participants with complete mental health assessments at both timepoints, but as sensitivity analysis we also repeated the clustering analysis including those with complete baseline SDQ data and noted no major differences between the number of clusters identified and their baseline profiles.

#### Clustering

SDQ scores were used to identify subgroups of adolescents with common profiles of mental health separately for each timepoint. Participants without mental health problems were allocated to a ‘non‐elevated difficulties’ (NED) group on the basis that they had SDQ scores in the ‘close to average range’ (Figure S1). At T1, 520 participants were assigned to the NED group and 784 participants with elevated difficulties were included in the clustering. At T2, 649 were identified as having NED, leaving 655 with elevated difficulties for clustering.

To optimise the clustering performance (Dalmaijer et al., 2022), data for those with elevated difficulties was reduced using uniform manifold approximation and projection (UMAP, see Supporting Information S1). For UMAP, the number of neighbours was set at 20 and the minimum distance at 0.0001 to allow for denser clusters and cleaner separations. K‐means clustering (*k* = 2–15) was then applied to the UMAP‐reduced space. The optimal number of clusters was chosen based on silhouette scores >0.5 (Basten et al., 2016; Robbins & Sahakian, 2003).

##### Cluster characterisation

Chi‐square tests were used to compare the proportion of males and females in each cluster at each timepoint to the proportion of males and females in the whole sample. Planned comparisons via pairwise Welch *t*‐tests (accounting for differences in sample size) were then used to compare the SDQ and cognitive scores between each cluster and the NED group.

##### Cluster transitions

We explored the number of adolescents who moved between any of the derived clusters and the NED group between T1 and T2. To evaluate whether transitions were statistically significant, observed proportions were compared to chance (i.e., equal transitions to each group). A series of planned comparisons via pairwise Welch *t*‐tests were used to characterise the T1 cognitive functioning of three types of transitions: (1) persisting—individuals who had difficulties across T1 and T2; (2) resolving—those who had difficulties at T1 but who transitioned to the NED group at T2; (3) emerging—those who were in the NED group at T1 but had difficulties at T2. The classification of persisting, resolving and emerging difficulties was completed for simple transitions (i.e., transitions from any of the data‐driven clusters to the NED group and vice‐versa, see Supporting Information S1) and for cluster‐specific transitions. For simple transitions, cluster membership was not used to differentiate specific profiles of mental health difficulties. For cluster‐specific transitions, changes in the type of mental health profile were considered; we explored whether the profiles were the same (homotypic) or different (heterotypic) over time. To pre‐empt the results, a three‐cluster solution was optimal at both timepoints, so for ease of interpretation, we report the planned comparisons based on a 3‐cluster solution at both T1 and T2 in Table 1.

**TABLE 1:** Planned comparisons to explore cognitive function linked to persisting, resolving, or emerging mental health problems.

	Transition	Compared to
Cluster‐specific transitions		
Emerging	NED at T1 moves to Cluster 1 at T2	NED at T1 and T2
	NED at T1 move to Cluster 2 at T2	NED at T1 and T2
	NED at T1 moves to Cluster 3 at T2	NED at T1 and T2
Persisting	Stays in Cluster 1 T1 to T2	NED at T1 and T2
	Stays in Cluster 2 T1 to T2	NED at T1 and T2
	Stays in Cluster 3 T1 to T2	NED at T1 and T2
Resolving	Cluster 1 at T1 moves to NED at T2	Stays in Cluster 1 T1 to T2
	Cluster 2 at T1 moves to NED at T2	Stays in Cluster 2 T1 to T2
	Cluster 3 at T1 moves NED at T2	Stays in Cluster 2 T1 to T2
Simple transitions		
Emerging	NED at T1 moves to Cluster 1, 2 or 3 at T2	NED at T1 and T2
Persisting	Stays in Cluster 1, 2 or 3 T1 to T2	NED at T1 and T2
Resolving	Cluster 1,2 or 3 at T1 moves to NED at T2	Stays in Cluster 1,2, or 3 T1 to T2

Abbreviation: NED, non‐elevated difficulties.

## RESULTS

Descriptives summarising the SDQ and cognitive task performance data for the sample at both timepoints is provided in Table S1.

### Clustering

UMAP data reduction combined with k‐means (*k* = 2–15) clustering was applied to the SDQ data separately at T1 and at T2 for all participants with scores outside the average range. Those with scores in the average range formed the NED group.

#### T1 clusters

**FIGURE 1:**
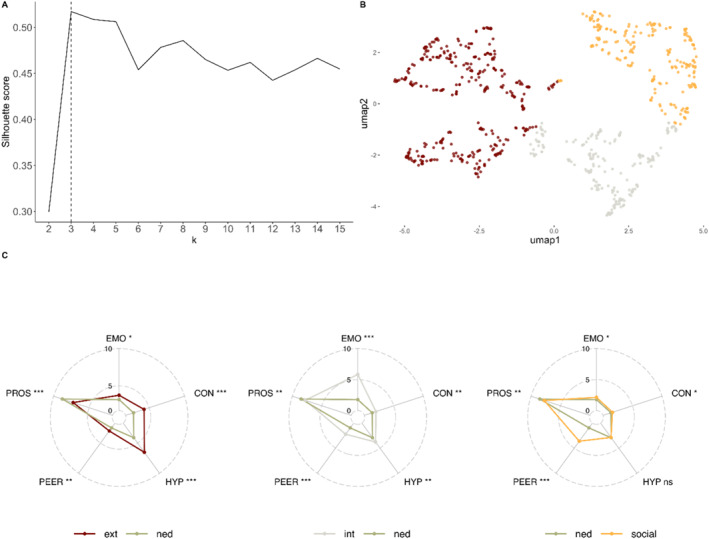
T1 cluster analysis and profiles of the three data‐driven clusters across SDQ subscales. (A) Silhouette scores of *k* = 2–15 cluster solutions at T1. (B) UMAP space showing a 2D projection of each participant's SDQ scores at T1. (C) SDQ profile of each cluster relative to the non‐elevated difficulties group at T1. Group differences are shown as effect sizes (* small [>0.2 and <0.5], ** medium [>0.5 and <0.8], *** large [>0.8]). With the exception of the PROS, higher scores indicate more difficulties. CON, conduct problems; EMO, emotional problems; ext, externalising profile; HYP, hyperactivity/inattention; int, internalising profile; NED, non‐elevated difficulties group; ns, non‐significant; PEER, peer problems; PROS, prosocial behaviour; SDQ, Strengths & Difficulties Questionnaire; UMAP, uniform manifold approximation and projection.

The T1 clustering results are presented in Figure 1. Figure 1A shows the average silhouette for different cluster solutions, indicating that a three‐cluster solution was optimal. This was further supported by 10‐fold cross‐validation, where the three‐cluster solution achieved the highest average silhouette score across folds (*M* = 0.51), outperforming all other solutions (*M* < 0.5). The reduced 2D UMAP space on which each participant's SDQ‐derived scores were projected is presented in Figure 1B.

The SDQ profiles of the three clusters are shown in Figure 1C. SDQ scores are compared to the NED group in each case and represented as effect sizes (Table S3). There were significant difficulties across most SDQ subscales for each cluster relative to the NED group, with specific areas of more severe problems on some subscales distinguishing each cluster from the others.

Relative to the NED group, the first cluster had significantly more difficulties across all SDQ subscales but had the most pronounced problems with hyperactivity (*d* = 1.73), conduct (*d* = 1.41), and prosocial behaviour (*d* = −1.34). As these problems represent overt behavioural/mental health difficulties, and because the hyperactivity and conduct subscales form the SDQ externalising index (Goodman et al., 2010), this profile was labelled Externalising (Ext.). The second cluster also had significantly more severe problems across all SDQ subscales compared to the NED group, with relatively more severe emotional (*d* = 3.10) and peer relationships (*d* = 1.12) problems. The sum of these two scales is used to index internalising problems (Goodman et al., 2010), so this cluster was labelled Internalising (Int.). The third cluster had significantly more difficulties than the NED group on all subscales, except Hyperactivity. The most pronounced difficulties for this cluster were in making/sustaining friendships (Peer Relationships; *d* = 2.66), so it was labelled Social. The NED group was the largest, followed by those with an externalising profile. Males were significantly overrepresented in the externalising and social clusters (*p* < .001), and females in the internalising cluster (*p* < .001; Table 2).

**TABLE 2:** Chi‐square results comparing the number of male and female participants in the non‐elevated difficulties group and the data‐driven clusters at T1.

Cluster at T1	Boys	Girls	*χ* ^2^	*p*
Internalising	48	151	41.31	<.001
Externalising	204	153	15.17	<.001
Social difficulties	136	92	14.99	<.001
Non‐elevated difficulties	223	297	3.29	.07

**FIGURE 2:**
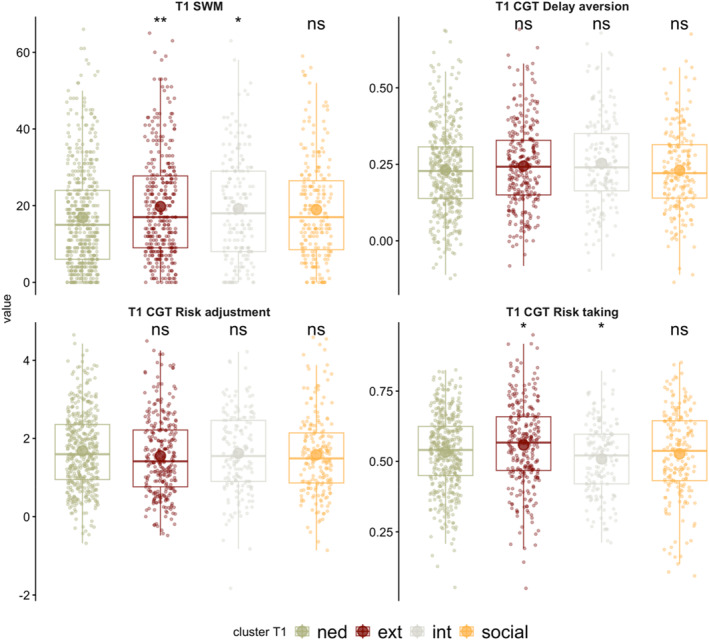
Cognitive profiles at T1 by cluster. Each cluster is compared to the non‐elevated difficulties group. Results based on two‐tailed Welch *t*‐tests. *p < .05, **p < .01. CGT, Cambridge gambling task; ext, externalising profile; int, internalising profile; NED, non‐elevated difficulties group; ns, non‐significant; SWM, spatial working memory.

The cognitive profiles of the T1 clusters were compared to the NED group (Figure 2 and Table S4). Those in the externalising cluster had significantly poorer SWM (*p* = .003) and significantly higher risk‐taking scores (*p* = .02) than the NED group. Those in the internalising cluster had significantly poorer SWM (*p* = .04) but were significantly less likely to take risks than the NED group (*p* = .04). There were no significant cognitive differences between those in the social cluster and the NED group.

#### T2 clusters

**FIGURE 3:**
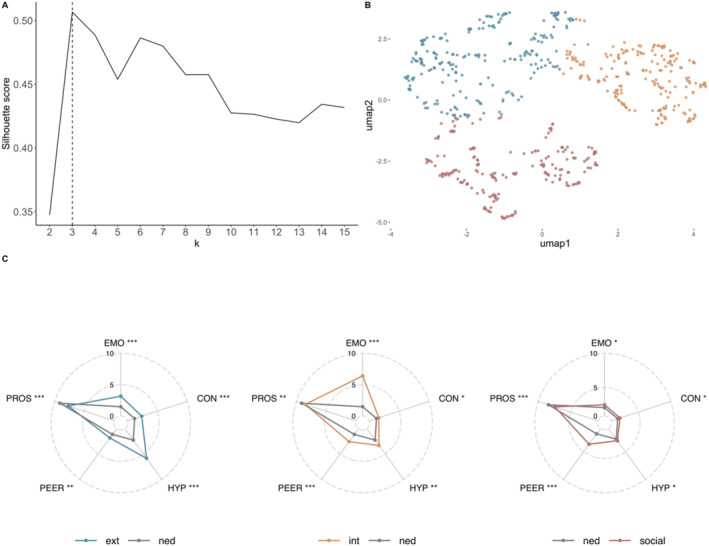
T2 cluster analysis and profiles of the three data‐driven clusters across SDQ subscales. (A) Silhouette scores of *k* = 2–15 cluster solutions at T2. (B) UMAP space showing a 2D projection of each participant's SDQ scores at T2. (C) SDQ profile of each cluster relative to the NED group at T2. Group differences are shown as effect sizes (* small [>0.2 and <0.5], ** medium [>0.05 and <0.8], *** large [>0.8]). Note that with the exception of the PROS scores higher scores indicate more difficulties. CON, conduct problems; EMO, emotional problems; ext, externalising profile; HYP, hyperactivity/inattention; int, internalising profile; NED, non‐elevated difficulties group; PEER, peer problems; PROS, prosocial behaviour; SDQ, Strengths & Difficulties Questionnaire; UMAP, uniform manifold approximation and projection.

K‐means clustering at T2 indicated that a three‐cluster solution was optimal (Figure 3A). This was further supported by 10‐fold cross‐validation, where the three‐cluster solution achieved the highest average silhouette score across folds (*M* = 0.51), outperforming all other solutions (*M* < 0.5). The 2D UMAP space on which SDQ‐derived scores were projected is presented in Figure 3B. While a 3‐cluster solution was optimal at both timepoints, the same participants did not belong to the same clusters at T1 and T2 (see Cluster Characterisations and Transitions below) and the profiles of the clusters changed subtly between timepoints.

SDQ profiles for T2 clusters are shown in Figure 3C. SDQ scores are compared to the NED group in each case and represented as effect sizes (Table S5). The profiles of the three clusters resembled those at T1. The first had significant difficulties across all subscales of the SDQ relative to the NED group and was characterised by relatively more pronounced problems with hyperactivity (*d* = 2.20), conduct (*d* = 1.15) and prosocial behaviour (*d* = −1.13). As two of these areas form the externalising problems index of the SDQ (Goodman et al., 2010), this profile was labelled Externalising (Ext.). At T2, this cluster also had pronounced emotional difficulties (*d* = 1.03). The second cluster also had significantly more severe problems across all subscales of the SDQ compared to the NED group, with relatively more severe emotional (*d* = 3.64) and peer relationships (*d* = 1.50) problems. This profile matched that of the second cluster at T1 and was similarly labelled Internalising (Int.). The third cluster was also characterised by significantly more problems across all subscales of the SDQ relative to the NED group, with the most pronounced areas of difficulty being with peer relationships (*d* = 2.46) and prosocial behaviour (*d* = −0.97), so as at T1, it was labelled Social. Table 3 shows that at T2 males were significantly overrepresented in the externalising cluster, and females in the internalising cluster (*p*s < .001); there were no significant differences in the numbers of males and females in the social cluster (*p* = .05). The NED group was the largest group, and this was larger at T2 than at T1 (*n* = 520 vs. *n* = 649, *χ* ^2^(1) = 52.23, *p* < .001). The discrepancies in the size of the internalising and externalising clusters were reduced at T2 (T1 N ext:int = 357:199, T2 N ext:int = 232:213, *χ* ^2^(3) = 78.18, *p* < .001), mostly due to there being fewer participants with an externalising profile at T2 (T1 *N* = 357, T2 *N* = 232, *χ* ^2^(1) = 60.27, *p* < .001).

**TABLE 3:** Chi‐square results comparing the number of male and female participants in the non‐elevated difficulties group and the data‐driven clusters at T2.

Group identity at T2	Boys	Girls	*χ* ^2^	*p*
Internalising	50	163	45.88	.001
Externalising	133	99	10.68	.001
Social difficulties	112	98	3.8	.05
Non‐elevated difficulties	313	336	0.67	.41

**FIGURE 4:**
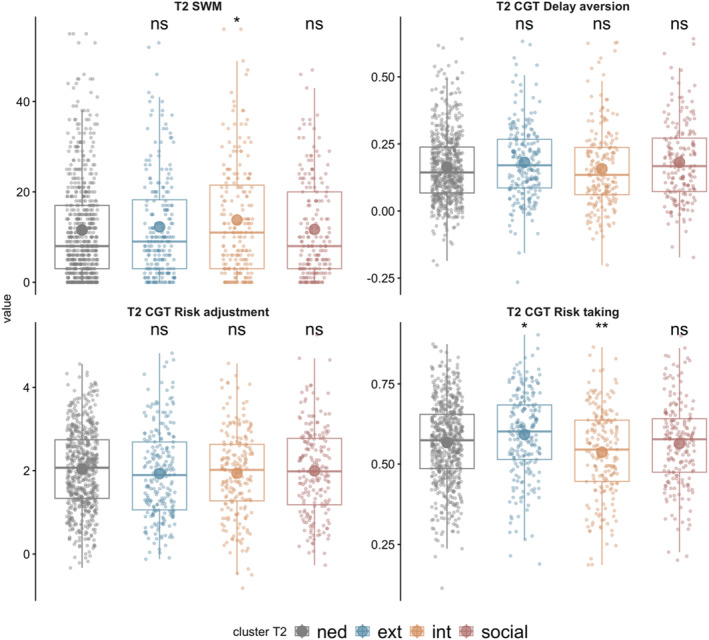
Cognitive profiles at T2 by cluster. Each cluster is compared to the non‐elevated difficulties group. Results based on two‐tailed Welch *t*‐tests. *p < .05, **p < .01. CGT, Cambridge gambling task; ext, externalising profile; int, internalising profile; NED, non‐elevated difficulties group; ns, non‐significant; SWM, spatial working memory.

Cognitive performance for the T2 clusters is presented in Figure 4 and Table S6. Those in the externalising cluster were significantly more likely to take risks than those in the NED group (*p* = .01), but there were no other significant group differences. Identical to the internalising cluster at T1, those in the internalising cluster at T2 were significantly less likely to take risks (*p* = .01) and had significantly poorer SWM (*p* = .03) relative to the NED group. No significant cognitive differences were observed between those in the social cluster and NED group.

### Transitions

Cluster‐specific transitions in mental health profiles were mapped between T1 and T2 (see Supporting Information S1 for simple transitions, Tables S10 and S11, and Figure S2). The proportion of adolescents transitioning across groups between T1 and T2 is shown in Table 4 (*p*‐values denote whether transitions are significantly different from chance i.e., assuming an equal chance of transitioning to any of the groups at T2). Most adolescents with NED at T1 continued to have NED at T2 (64%). For each data‐driven mental health cluster at T1, the most likely transition was to the NED group at T2 (across clusters ∼40%), followed by having the same profile at both T1 and T2 (∼30%).

**TABLE 4:** Cluster‐specific transitions between mid‐ (T1) and late (T2) adolescence.

Cluster T1	Cluster T2	*N*	%	*p*
Ext.	Ext.	113	32	.004
	Int.	54	15	<.001
	NED	141	39	<.001
	Social	49	14	<.001
Int.	Ext.	32	16	.005
	Int.	63	32	.04
	NED	76	38	<.001
	Social	28	14	.001
ND	Ext.	59	11	<.001
	Int.	65	12	<.001
	NED	333	64	<.001
	Social	63	12	<.001
Social	Ext.	28	12	<.001
	Int.	31	14	<.001
	NED	99	43	<.001
	Social	70	31	.06

Note: p‐values denote chi‐square results testing the hypothesis that the proportion transitioning into each cluster is equal to chance.

Abbreviations: Ext., externalising profile; Int., internalising profile; NED, non‐elevated difficulties group.

**FIGURE 5:**
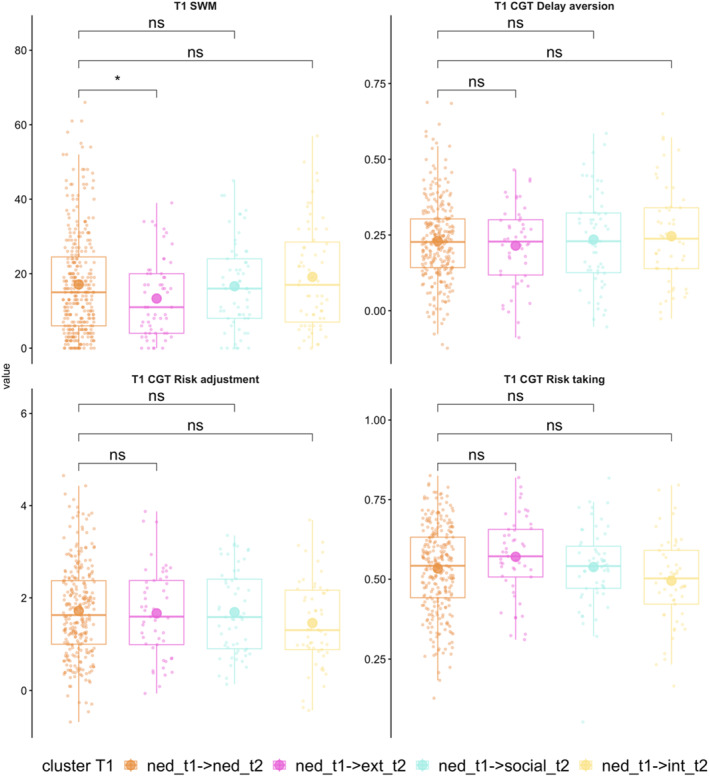
Differences in T1 cognitive performance for those with different profiles of emerging mental health problems relative to the stable non‐elevated difficulties group. Results based on two‐tailed Welch *t*‐tests. *p < .05, **p < .01. CGT, Cambridge gambling task; ext, externalising profile; int, internalising profile; NED, non‐elevated difficulties group; ns, non‐significant; soc, social profile; SWM, spatial working memory.

To explore the relationship between changes in mental health over time and cognitive function, the transitions between T1 and T2 were classified as either persisting, emerging, or resolving. There were three groups with emerging difficulties: (i) those with NED at T1 and a profile of externalising problems at T2; (ii) those with NED at T1 and a profile of internalising problems at T2; (iii) those with NED at T1 and a profile of social difficulties at T2. The cognitive function of each of these groups at T1 was compared to the baseline cognitive function of the group who had NED across time in a series of planned pairwise Welch *t*‐tests (Figure 5 and Table S7). For the most part, there were no significant group differences except for those who developed externalising problems having better baseline SWM than those with NED.

**FIGURE 6:**
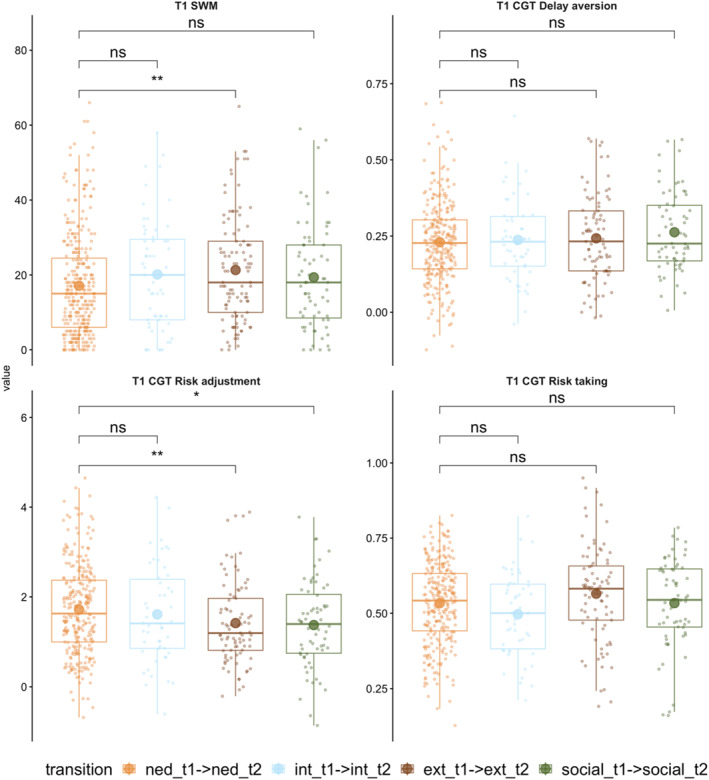
Differences in T1 cognitive performance for those with persisting mental health problems that do not change in profile over time relative to the stable non‐elevated difficulties group. Results based on two‐tailed Welch *t*‐tests. *p < .05, **p < .01. CGT, Cambridge gambling task; ext, externalising profile; int, internalising profile; NED, non‐elevated difficulties group; ns, non‐significant; SWM, spatial working memory.

There were three groups with persisting difficulties across time, classified so because their profile of problems did not change between T1 and T2 (e.g., a profile of persisting predominantly externalising, internalising, or social problems). The baseline cognitive function of each of these groups was compared to that of the group with NED across T1 and T2 (see Figure 6 and Table S8). Those with persistent externalising symptoms had poorer SWM and were less able to adjust their risk‐related behaviour than those who were in the stable NED group. Those who had a profile of persistent difficulties with social relationships were also significantly less able to adjust their risk‐related behaviour compared to those who had an NED profile over time. There were no other significant group differences.

**FIGURE 7:**
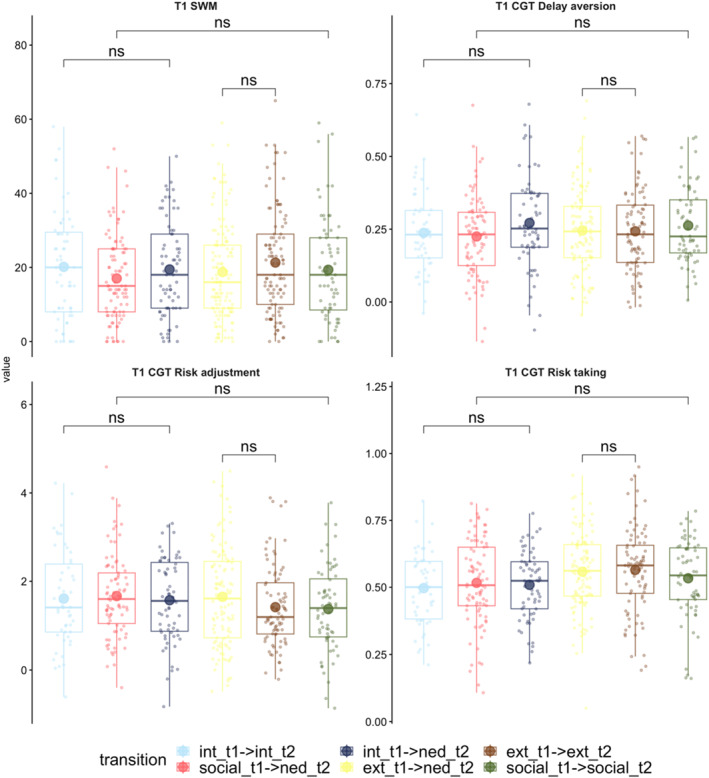
Differences in T1 cognitive performance for those with resolving mental health problems compared to those with problems that persist and do not change in profile over time. Results based on two‐tailed Welch *t*‐tests. *p < .05, **p < .01. CGT, Cambridge gambling task; ext, externalising profile; int, internalising profile; NED, non‐elevated difficulties group; ns, non‐significant; SWM, spatial working memory.

The third category of transitions, resolving, also had three groups. Each group's baseline cognitive function was compared to that of a group that had persisting difficulties in the area in which those with resolved problems had difficulties at T1. The comparisons were therefore: (i) those with externalising problems at T1 who moved to the NED group at T2 compared to those with persisting externalising problems at T1 and T2; (ii) those with internalising problems who moved to the NED group at T2 compared to those with persisting problems at T1 and T2; and (iii) those with social problems at T1 who moved to the NED group at T2 compared to those with persisting social problems at T2. There were no significant group differences (see Figure 7 and Table S9).

## DISCUSSION

Using a data‐driven transdiagnostic clustering approach we identified three distinct profiles of mental health difficulties in a sample of over 1000 adolescents. One with relatively more severe externalising symptoms, another with relatively more pronounced internalising problems, and a third with difficulties with social relationships. The same profiles emerged during mid‐ and late adolescence, with only subtle differences in symptom profiles across time. Mental health profiles changed over time for some participants. Difficulty adjusting risk‐related behaviour was linked to persistent externalising and social problems from mid‐ to late adolescence and poor SWM to persistent externalising problems.

### Mental health profiles

The three profiles identified at both timepoints had broad‐ranging difficulties relative to a subgroup with no mental health symptoms but were distinguished from one another by different profiles of either predominantly externalising, internalising, or social problems. These cluster profiles are similar to those identified in 5‐ and 17‐year‐olds in another sample drawn from the Millenium Cohort Study data (Benhamou et al., 2025). In Benhamou et al.'s (2025) study, cluster profiles corresponding to emotion, conduct and social were identified, which broadly correspond to the internalising, externalising and social cluster profiles identified in the current study. Together these studies suggest the profiles of mental health difficulties that emerge as young as 5 years are present in mid‐ and late adolescence. These profiles also align with dimensions identified in contemporary hierarchical models of psychopathology (e.g., the Hierarchical Taxonomy of Psychopathology (Kotov et al., 2017)), which include broad internalising and externalising factors corresponding to the internalising and externalising clusters found here. These higher‐level factors break down into lower‐order dimensions, one of which, ‘social maladjustment’ (Holmes et al., 2021), resembles our social cluster. Our clustering therefore aligns with dimensional models and further complements them by enabling us to plot an individual's position in multidimensional space and their stability and changes across developmental time.

#### Cluster 1—Externalising

In mid‐adolescence, this profile was associated with relatively more severe conduct problems, elevated levels of hyperactivity, and more antisocial behaviour. By late adolescence, it also included elevated emotional problems. This reflects trends observed in other studies showing early externalising symptoms predict later internalising symptoms (Dugré & Potvin, 2022). At both timepoints, this profile was associated with increased risk‐taking, consistent with studies showing difficulties inhibiting impulsive responses and delaying gratification in adolescence are associated both with risk‐taking and increased vulnerabilities to elevated externalising symptoms (Dumontheil, 2016).

#### Cluster 2—Internalising

This profile, characterised by pronounced difficulties with emotional problems and peer relationships, did not change between mid‐ and late adolescence: at both timepoints, individuals in this cluster had relatively more severe internalising problems, accompanied by poorer working memory and increased risk aversion relative to adolescents with no mental health difficulties. Associations between poor working memory and internalising symptoms in adolescence may reflect either the negative impact of anxious or depressive symptoms on the limited resources of working memory (Owens et al., 2012), or the impact of poor working memory on the ability to direct attention away from worrisome or negative thoughts (Koster et al., 2017). Existing evidence for associations between altered risk‐taking behaviour and internalising symptoms in adolescence is mixed, typically finding no links or positive correlations (Prendergast et al., 2019; Spriggs & Halpern, 2008). Finding that risk‐taking was lower in adolescents with a predominantly internalising profile suggests they may exercise more caution and require more certainty when making decisions, which may reflect more general patterns of avoidant behaviour associated with internalising symptoms (Peris & Galván, 2021).

#### Cluster 3—Social

This profile was characterised by relatively more severe problems with peer relationships at both timepoints, accompanied by lower levels of prosocial behaviour in late adolescence. There were no differences in cognitive function between adolescents with this profile and those with no mental health problems. This could be because profiles of pronounced social problems arise through social or environmental factors.

### Prevalence

Contrary to evidence suggesting mental health problems increase into late adolescence (Beauchamp et al., 2018) our data revealed there were fewer individuals with mental health difficulties in late‐ adolescence. This likely reflects the lack of follow‐up data from participants with more pronounced mental health symptoms at baseline. Consistent with previous reports males were overrepresented in the externalising and females in the internalising subgroup at both timepoints (Humphreys et al., 2019).

### Transitions

Transitions between clusters were similar for each of the three profiles: mental health problems resolved for ∼40% of adolescents; ∼30% of each cluster had a similar profile of mental health problems across time, and the remaining 30% transitioned to one of the other two profiles, in each case with a reasonably even split between the clusters into which they moved.

In mid‐adolescence, the externalising cluster was larger relative to the internalising or social clusters. In late adolescence, the distribution was more balanced, primarily because there was a decrease in the number of adolescents experiencing predominantly externalising problems. This reduction is consistent with Bathelt et al.'s (2021) study showing that externalising symptoms reduce with age. The proportion of adolescents in the internalising and social clusters was similar across time.

Transitions were classified as either emerging or resolving in late adolescence or persisting across time. Both SWM and the ability to adjust risk‐related behaviours in mid‐adolescence were related to problems that persisted, but not to problems that resolved or emerged over time. This effect was driven by persistent externalising problems, which were related to both poorer SWM and risk adjustment problems at age 14, and by persistent social difficulties, which were related to poorer risk adjustment skills at 14 years.

Finding a specific link between working memory and persistent externalising problems is consistent with the literature (Huang‐Pollock et al., 2017; Mareva & Holmes, 2019). Working memory may contribute to the maintenance of externalising symptoms because it indexes executive functions that play an important role in regulating and controlling behaviour (Nigg et al., 2006). Risk adjustment was linked to persistent externalising and socialising difficulties, but risk‐taking was not. This runs counter to evidence suggesting risk‐taking increases vulnerability to mental health difficulties during adolescence (Dumontheil, 2016). It is, however, consistent with the notion that adolescent risk‐taking can be adaptive and useful in the transition to adulthood and in peer socialisation during adolescence, and that it is only those who engage in risky behaviour without sufficient cognitive control (i.e., without the capacity to adjust risk‐related behaviours) who are vulnerable to adverse outcomes (Romer et al., 2011).

There was no association between baseline cognitive function and mental health symptoms that resolved over time. Other factors such as leaving education and moving into employment might contribute to these changes. The absence of an association between baseline cognitive skills and emerging mental health problems suggests that good cognitive function in mid‐adolescence does not necessarily protect against later mental health problems, and likewise, that poor cognitive function does not increase vulnerability to the onset of later mental health problems. This is an unexpected finding given the wealth of literature implicating cognitive difficulties in the onset and maintenance of mental health difficulties (LeMoult & Gotlib, 2019). One explanation is that the impact of cognitive variability on the onset of mental health symptoms between mid‐ and late adolescence is washed out by other more impactful factors such as increased stress as individuals take on adult roles and responsibilities.

### Limitations

There are several limitations to the current study. First, the range of mental health symptoms and cognitive assessments was limited to those available at both timepoints. This limits the range of profiles detectable and the depth with which we could explore links to cognition. Second, although the IMAGEN cohort is designed to be population‐representative, participants receiving mental health treatment were excluded which may have introduced bias into our results. Second, and relatedly, the sample size was relatively low for clustering. While sample size does not increase power in cluster analyses, replication of these analyses in larger samples is encouraged. On this note, confidence in our results is provided by the similarity in cluster profiles between those identified in 5‐ and 17‐year‐olds in the much larger Millenium Cohort Study data. Benhamou et al. (2025) identified cluster profiles corresponding to emotion, conduct and social that are broadly similar to the internalising, externalising and social cluster profiles identified in the current study. Third, our analyses were based on self‐report data. Obtaining data from other informants may have produced different results. Fourth, we did not consider the different combinations of interacting genetic, biological, and environmental factors that may have shaped the relationship between cognition and mental health; this would be a fruitful avenue for future investigations. Fifth, our analyses were based on self‐report data. Obtaining data from other informants may have produced different results. Sixth, there were multiple permutations for capturing transitions, so even though our sample size was large, we had to pre‐plan which comparisons to focus on when comparing the mental health and cognitive profiles of the clusters, As a result we only compared the profile of each cluster to the NED group, rather than comparing each pairwise combinations of clusters to avoid issues with multiple comparisons. Finally, K‐means clustering was chosen as it replicates Benhamou's et al. (2025) approach and because its flexible algorithm makes fewer assumptions about the data's underlying distribution relative to alternatives such as growth mixture modelling. Nonetheless, we acknowledge that analytic approaches that model the joint developmental trajectories of co‐occurring symptoms would provide further insights for these data.

## CONCLUSION

A data‐driven clustering approach identified profiles of mental health problems in mid‐and late adolescence that were similar over time. One was linked to predominantly externalising problems and elevated risk‐taking, with an over‐representation of males. The second had relatively more severe internalising problems, accompanied by poor working memory and an aversion to risk, and was more common in females. The third had difficulties with social relationships and no observable cognitive problems. Exploring transitions over time revealed that externalising problems were more common in mid‐adolescence. Persistent externalising and social problems were related to cognitive function in mid‐adolescence, but problems that emerged or resolved in late adolescence were not. This is the first study of its kind to map transitions in mental health profiles between mid‐ and late adolescence. Understanding more about what predicts these shifts will enable us to move towards proactive models of mental health support.

## AUTHOR CONTRIBUTIONS

**Silvana Mareva:** Conceptualization; formal analysis; methodology; project administration; supervision; writing—original draft; writing—review and editing. **Jenna Parker:** Conceptualization; formal analysis; methodology; project administration; writing—original draft; writing—review and editing. **Marc Bennett:** Conceptualization; supervision; writing—original draft; writing—review and editing. **Laura Pass:** Supervision; writing—review and editing. **Tobias Banaschewski:** Data curation. **Arun L. W. Bokde:** Data curation. **Sylvane Desrivières:** Data curation. **Herta Flor:** Data curation. **Sarah Hohmann:** Data curation. **Dimitri Papadopoulos Orfanos:** Data curation. **Hugh Garavan:** Data curation. **Penny Gowland:** Data curation. **Henrik Walter:** Data curation. **Rüdiger Brühl:** Data curation. **Jean‐Luc Martinot:** Data curation. **Marie‐Laure Paillère Martinot:** Data curation. **Eric Artiges:** Data curation. **Frauke Nees:** Data curation. **Tomáš Paus:** Data curation. **Luise Poustka:** Data curation. **Michael N. Smolka:** Data curation. **Nilakshi Vaidya:** Data curation. **Robert Whelan:** Data curation. **Gunter Schumann:** Data curation. **Joni Holmes:** Conceptualization; formal analysis; funding acquisition; investigation; methodology; project administration; supervision; writing—original draft; writing—review and editing.

## CONFLICT OF INTEREST STATEMENT

The authors declare no conflicts of interest.

## ETHICAl CONSIDERATIONS

The institutional ethics committee of King's College London (PNM/10/11‐126), University of Nottingham (D/11/2007), Trinity College Dublin (SPREC092007‐01), Technische Universitat Dresden (EK 235092007), Commissariat a l'Energie Atomique et aux Energies Alternatives, INSERM (2007‐A00778‐45), University Medical Center at the University of Hamburg (M‐191/07) and in Germany at medical ethics committee of the University of Heidelberg (2007‐024N‐MA) in accordance with the Declaration of Helsinki.

## Supporting information

Supporting Information S1

## Data Availability

The data that support the findings of this study are openly available https://www.imagen‐project.org/ at https://doi.org/10.25720/p1ma‐genq.
