# Supplementary material for: Mapping developmental transitions in mental health from mid‐ to late‐adolescence: Concurrent and longitudinal links to cognition
Source: JCPP Adv. 2025 Jun 5;6(1):e70016. doi: 10.1002/jcv2.70016 (PMC12973140; doi:10.1002/jcv2.70016)
Supplement: Supplementary file 1 — Supporting Information S1 [file JCV2-6-e70016-s001.docx]

**Supporting Information**

**Cognitive tasks**

**Cambridge Gambling Task (CGT).** The CGT was used to measure risk tasking and delay aversion. On each trial, participants were presented with a row of ten boxes across the top of the screen, some red and some blue. The ratio of red to blue boxes could range from 1 red: 9 blue, through to 1 blue: 9 red, with each red: blue-pair having an equal chance of occurring. Participants were first required to guess whether a yellow token was hidden in one of the red or blue boxes by clicking on a blue or red rectangle presented on screen. They started with a set number of points and were then required to bet a proportion of these on the confidence of their judgement. The bet proportions were fixed across trials at 5%, 25%, 50%, 75%, or 95%, and presented on screen sequentially with a fixed delay between each proportion, requiring participants to delay their responses to select their preferred bet. The presentation order (ascending or descending) of the bet proportions was varied across trials. Participants were rewarded with additional points for correct guesses, and lost points for incorrect guesses. Multiple indices captured different aspects of risk-taking.

In this study we used the measures of delay aversion /impulsivity, risk tasking, and risk adjustment as they reflect most closely the aspects of cognitive function linked to risky behaviour and mental health problems in adolescence [1–4]. Impulsivity / delay aversion was calculated as the difference in average betting ratios chosen across ascending and descending conditions for optimal trials (those where the participant chose the colour with the most boxes). Larger negative values (lower values) correspond to impulsive betting (e.g., choosing the bets that appear on screen sooner). Risk taking was measured as average of the proportion of points participants were willing to bet on optimal trials. Higher values corresponded to betting a higher proportion of points, indicating someone was better able to tolerate risks (e.g., they were less risk averse / more will to take risks). Risk adjustment was included to capture participant’s tendencies to bet more when the odds were in their favour, with lower scores representing poorer risk adjustment (e.g., being less likely to switch the choice of coloured box when the rules or ratio of blue: red boxes changed).

**Spatial Working Memory (SWM).** The SWM task was a self-ordered, serial search task, in which participants were presented with a set of boxes on screen and told to search for a token that was hidden beneath one of them. Participants searched the boxes by clicking on them one at a time. When the token had been found, participants were shown the same set of boxes and instructed to find the next token. They were told that the once a token had been found under a particular box, it would not appear under the same box again. Within a block of trials, each box would have the token hidden under it once. Thus, to perform the task well, participants had to remember which boxes the token had already been hidden under in any block of trials. The number of boxes on screen increased within a set of trials from 4 to 6 to 8, to increase the memory load (participants had to remember whether a token had been hidden under a larger number of boxes, across more trials). A new block of trials began once a participant had found the token under each box. The change in block was marked by a change in position and colour of the boxes. A higher score indicated more errors, suggesting poorer working memory. Each memory load condition (i.e., number of boxes on the screen) was presented four times (four trials with 4 boxes, four trials with 6 boxes, four trials with 8 boxes) resulting in a total 72 tokens that had to be located across 12 search sets. Between trial errors were recorded, representing the number of times a participant searched a box in which in a token had been found in an earlier trial within a block. The higher the score, the greater the number of errors (or memory failures), indicating poorer working memory.

**R-packages**

Analyses were completed using the R-packages psych 2.2.3 [5] and NbClust 3.0 [6].

**Table S1**

*Descriptive statistics for all SDQ subscales and cognitive tasks at both timepoints*

|  | T1: Baseline | | |  | T2: Follow-up | | |
| --- | --- | --- | --- | --- | --- | --- | --- |
| SDQ Scale | *N* | *M* | *SD* |  | *N* | *M* | *SD* |
|  |  |  |  |  |  |  |  |
| Emotional Problems | 1,304 | 2.65 | 2.06 |  | 1,304 | 2.66 | 2.37 |
| Conduct Problems | 1,304 | 1.97 | 1.43 |  | 1,304 | 1.55 | 1.17 |
| Peer Problems | 1,304 | 1.79 | 1.58 |  | 1,304 | 1.86 | 1.38 |
| Hyperactivity | 1,304 | 3.85 | 2.11 |  | 1,304 | 3.14 | 2.1 |
| Prosocial Behaviour | 1,304 | 7.78 | 1.63 |  | 1,304 | 8.56 | 1.54 |
| SWM Between errors | 1,248 | 18.36 | 13.52 |  | 1,213 | 12.07 | 11.51 |
| CGT Delay aversion | 1,074 | 0.24 | 0.14 |  | 1,216 | 0.17 | 0.14 |
| CGT Risk adjustment | 1,079 | 1.62 | 1 |  | 1,224 | 1.99 | 1.01 |
| CGT Risk taking | 1,079 | 0.54 | 0.14 |  | 1,223 | 0.57 | 0.13 |

*Note*. SWM = Spatial working memory; CGT = Cambridge Gambling Task

**Table S2**

*Comparison of SDQ data at T1 for participants with and without T2 data.*

|  | *N* | *M* | *SD* | *N* | *M* | *SD* | *t* | *df* | *p* | *d* |
| --- | --- | --- | --- | --- | --- | --- | --- | --- | --- | --- |
|  | Participants without T2 SDQ data | | | Participants with T2 SDQ data | | |  |  |  |  |
| Emotional problems | 872 | 2.64 | 2.05 | 1304 | 2.65 | 2.06 | -0.03 | 1870.57 | .98 | 0.001 |
| Conduct problems | 872 | 2.34 | 1.69 | 1304 | 1.97 | 1.43 | 5.32 | 1657.34 | <.001 | -0.24 |
| Hyperactivity | 872 | 4.30 | 2.20 | 1304 | 3.85 | 2.11 | 4.82 | 1812.66 | <.001 | -0.21 |
| Peer problems | 872 | 1.88 | 1.65 | 1304 | 1.79 | 1.58 | 1.28 | 1814.75 | .20 | -0.06 |
| Prosocial behaviour | 872 | 7.52 | 1.74 | 1304 | 7.78 | 1.63 | -3.44 | 1778.55 | .001 | 0.15 |

*Note.* Results are based on two-tailed Welch *t*-tests.

**UMAP**

To optimise clustering performance [7], data for those with elevated difficulties was reduced using uniform manifold approximation and projection (UMAP, see Supplement for details). UMAP initially builds a topological representation of the original data, before minimising the cross-entropy between the new lower-dimensional space and the original higher-dimensional space to optimise the lower-dimensional embedding. This method was favoured as it has been shown to outperform principal components analysis, multi-dimension scaling, and other data reduction methods for its ability to preserve non-linear variable interactions and the local and global structure of the original data, its flexibility regarding choice of distance metrics, its computational efficiency and its ability to reproduce results (Yan et al., 2021).

**Figure S1**

*SDQ Scoring Protocol***
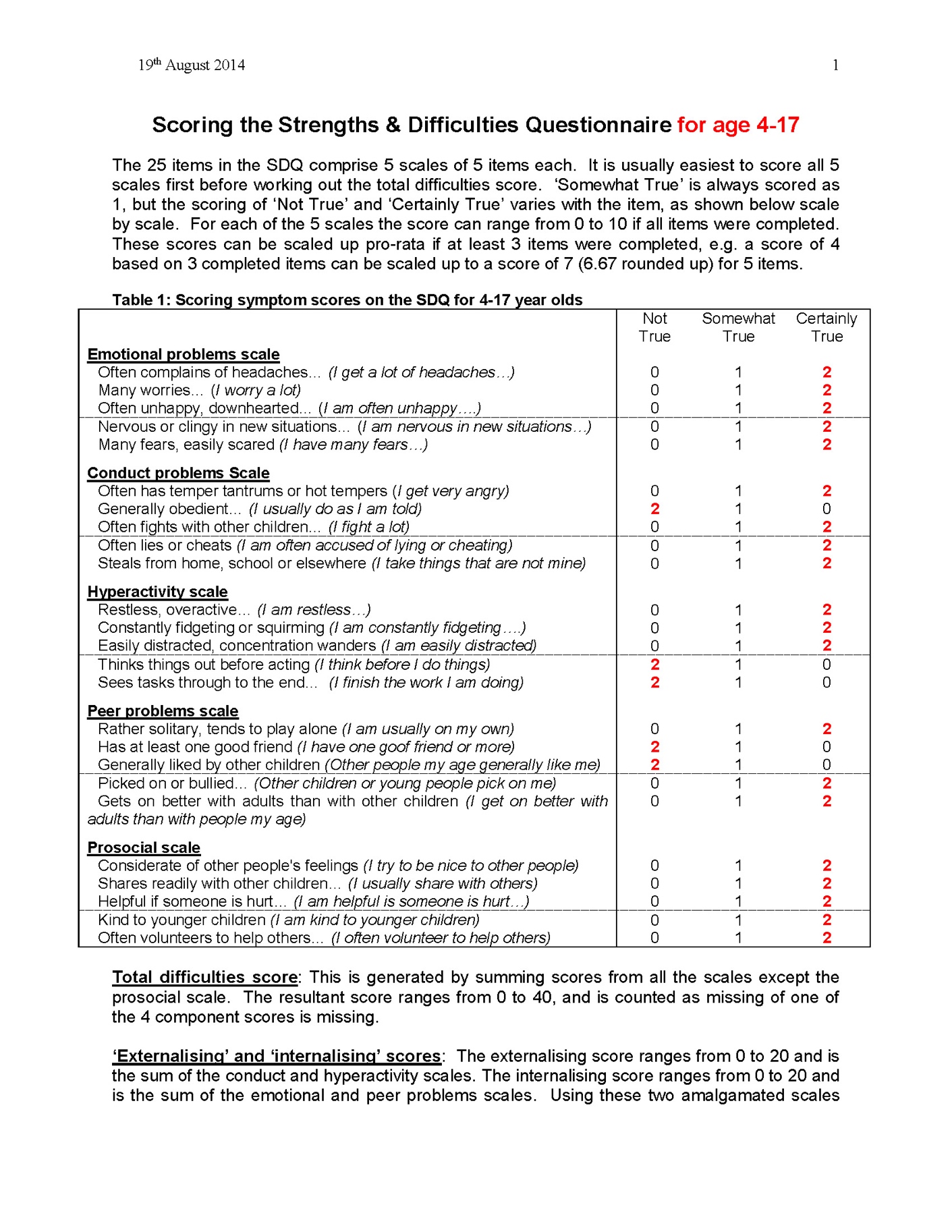
**

**
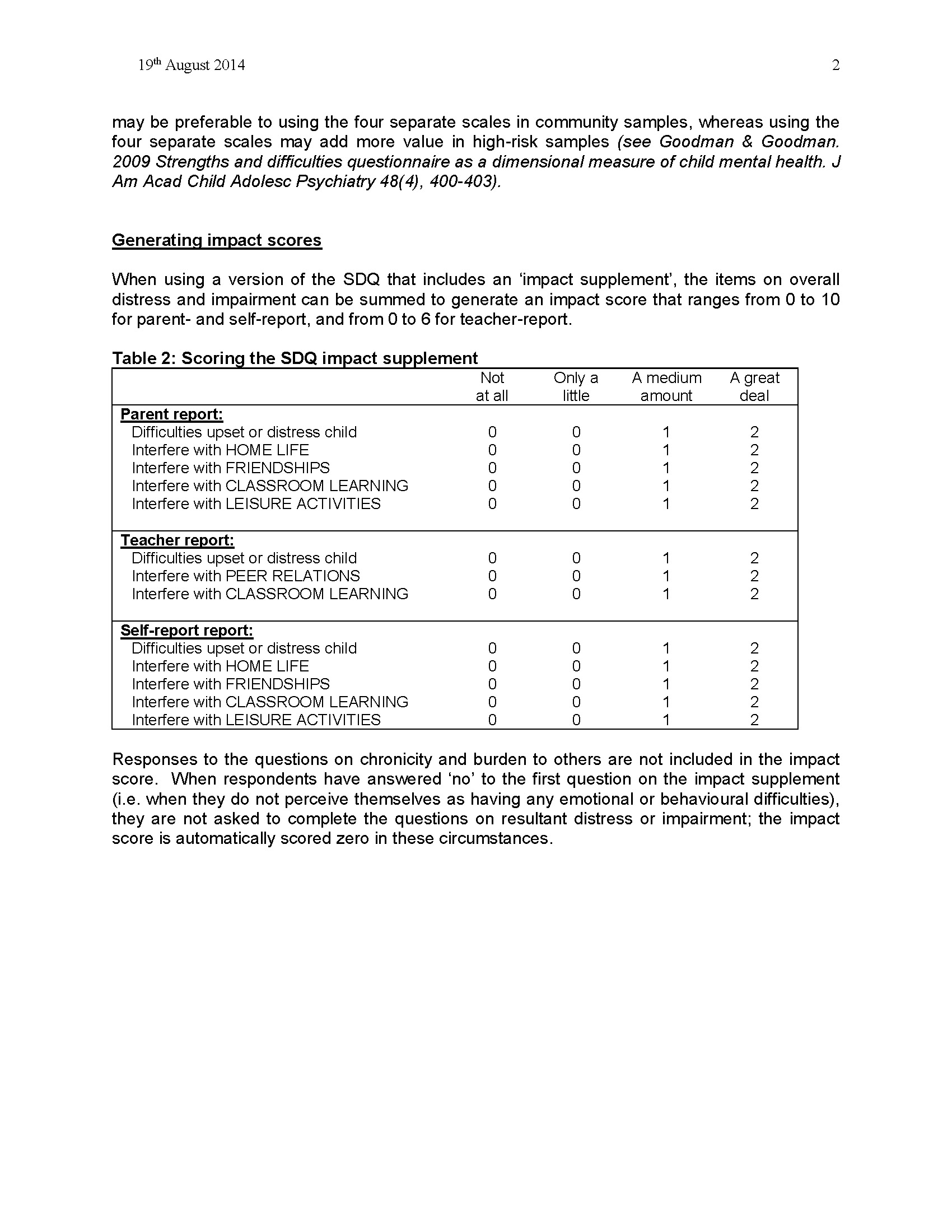

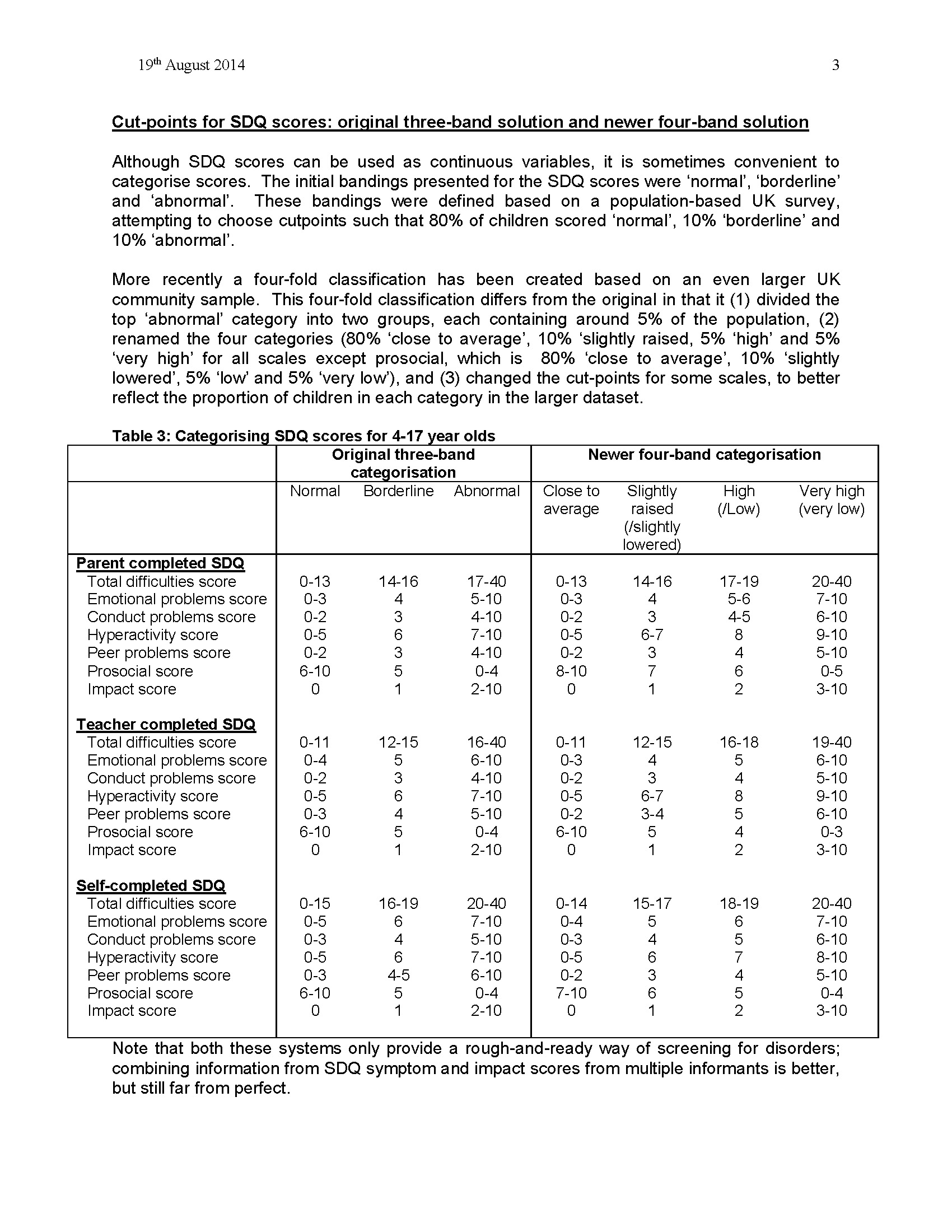
**

**Table S3**

*Comparison of SDQ scores across clusters at T1*

| SDQ subscale | *N* | *M* | *SD* | *N* | *M* | *SD* | *t* | *df* | *p* | *d* |
| --- | --- | --- | --- | --- | --- | --- | --- | --- | --- | --- |
| Cluster 1 [ext.] |  |  |  | Non-elevated difficulties | | |  |  |  |  |
| Emotion problems | 357 | 2.48 | 1.91 | 520 | 1.77 | 1.28 | -6.11 | 572.5 | <.001 | 0.45 |
| Conduct problems | 357 | 3.13 | 1.68 | 520 | 1.34 | 0.89 | -18.42 | 495.11 | <.001 | 1.41 |
| Hyperactivity | 357 | 5.83 | 1.82 | 520 | 2.93 | 1.57 | -24.47 | 687.31 | <.001 | 1.73 |
| Peer problems | 357 | 1.58 | 1.48 | 520 | 0.97 | 0.78 | -7.12 | 494.83 | <.001 | 0.54 |
| Prosocial behaviour | 357 | 6.69 | 1.77 | 520 | 8.52 | 0.99 | 17.67 | 508.88 | <.001 | -1.34 |
| Cluster 2 [int.] |  |  |  |  |  |  |  |  |  |  |
| Emotion problems | 199 | 5.83 | 1.39 | 520 | 1.77 | 1.28 | -35.87 | 334.22 | <.001 | 3.1 |
| Conduct problems | 199 | 1.98 | 1.26 | 520 | 1.34 | 0.89 | -6.48 | 276.65 | <.001 | 0.63 |
| Hyperactivity | 199 | 3.78 | 1.85 | 520 | 2.93 | 1.57 | -5.76 | 313.02 | <.001 | 0.52 |
| Peer problems | 199 | 2.23 | 1.73 | 520 | 0.97 | 0.78 | -9.88 | 229.95 | <.001 | 1.12 |
| Prosocial behaviour | 199 | 7.83 | 1.6 | 520 | 8.52 | 0.99 | 5.66 | 258.14 | <.001 | -0.58 |
| Cluster 3 [social] |  |  |  |  |  |  |  |  |  |  |
| Emotion problems | 228 | 2.13 | 1.46 | 520 | 1.77 | 1.28 | -3.22 | 385.77 | .001 | 0.27 |
| Conduct problems | 228 | 1.6 | 1.02 | 520 | 1.34 | 0.89 | -3.38 | 384.19 | .001 | 0.28 |
| Hyperactivity | 228 | 2.87 | 1.62 | 520 | 2.93 | 1.57 | 0.5 | 421.07 | .61 | -0.04 |
| Peer problems | 228 | 3.62 | 1.36 | 520 | 0.97 | 0.78 | -27.48 | 295.35 | <.001 | 2.66 |
| Prosocial behaviour | 228 | 7.76 | 1.63 | 520 | 8.52 | 0.99 | 6.52 | 303 | <.001 | -0.62 |

*Note.* Results based on two-tailed Welch *t*-tests. Ext. = externalising; Int. = internalising.

**Table S4**

*Cognitive profiles at T1 by cluster*

| Cognitive task | *N* | *M* | *SD* | *N* | *M* | *SD* | *t* | *df* | *p* | *d* |
| --- | --- | --- | --- | --- | --- | --- | --- | --- | --- | --- |
| Ext. cluster |  |  |  | Non-elevated difficulties | | |  |  |  |  |
| SWM Between errors | 338 | 19.76 | 13.8 | 505 | 16.87 | 13.35 | -3.03 | 707.68 | .003 | 0.21 |
| CGT Delay aversion | 280 | 0.24 | 0.14 | 435 | 0.23 | 0.14 | -1.26 | 606.25 | .21 | 0.10 |
| CGT Risk adjustment | 281 | 1.55 | 1.01 | 439 | 1.68 | 0.98 | 1.65 | 580.13 | .10 | -0.13 |
| CGT Risk taking | 281 | 0.56 | 0.15 | 439 | 0.53 | 0.13 | -2.27 | 549.15 | .02 | 0.18 |
| Int. cluster |  |  |  |  |  |  |  |  |  |  |
| SWM Between errors | 186 | 19.22 | 13.7 | 505 | 16.87 | 13.35 | -2.02 | 323.28 | .04 | 0.17 |
| CGT Delay aversion | 163 | 0.25 | 0.15 | 435 | 0.23 | 0.14 | -1.63 | 276.68 | .10 | 0.15 |
| CGT Risk adjustment | 163 | 1.62 | 1.03 | 439 | 1.68 | 0.98 | 0.65 | 277.4 | .52 | -0.06 |
| CGT Risk taking | 163 | 0.51 | 0.13 | 439 | 0.53 | 0.13 | 2.1 | 299.61 | .04 | -0.19 |
| Social cluster |  |  |  |  |  |  |  |  |  |  |
| SWM Between errors | 219 | 18.94 | 13.2 | 505 | 16.87 | 13.35 | -1.94 | 419.56 | .05 | 0.16 |
| CGT Delay aversion | 196 | 0.23 | 0.14 | 435 | 0.23 | 0.14 | 0.04 | 385.54 | .97 | -0.003 |
| CGT Risk adjustment | 196 | 1.58 | 1.03 | 439 | 1.68 | 0.98 | 1.11 | 355.96 | .27 | -0.1 |
| CGT Risk taking | 196 | 0.53 | 0.15 | 439 | 0.53 | 0.13 | 0.52 | 329.71 | .6 | -0.05 |

*Note*. SWM = Spatial working memory; CGT = Cambridge Gambling Task; Ext. = externalising; Int. = internalising. Results based on two-tailed Welch *t*-tests.

**Table S5**

*Comparison of SDQ scores across clusters at T2*

| SDQ subscale | *N* | *M* | *SD* | *N* | *M* | *SD* | *t* | *df* | *p* | *d* |
| --- | --- | --- | --- | --- | --- | --- | --- | --- | --- | --- |
| Cluster 1 [ext.] |  |  |  | Non-elevated difficulties | | |  |  |  |  |
| Emotion problems | 232 | 3.16 | 2.25 | 649 | 1.48 | 1.34 | -10.74 | 291.87 | <.001 | 1.03 |
| Conduct problems | 232 | 2.39 | 1.5 | 649 | 1.21 | 0.81 | -11.43 | 279.93 | <.001 | 1.15 |
| Hyperactivity | 232 | 5.86 | 1.77 | 649 | 2.27 | 1.59 | -27.22 | 371.16 | <.001 | 2.2 |
| Peer problems | 232 | 1.85 | 1.39 | 649 | 1.14 | 0.77 | -7.38 | 283.32 | <.001 | 0.73 |
| Prosocial behaviour | 232 | 7.68 | 1.8 | 649 | 9.11 | 1.03 | 11.54 | 286.8 | <.001 | -1.13 |
| Cluster 2 [int.] |  |  |  |  |  |  |  |  |  |  |
| Emotion problems | 213 | 6.41 | 1.39 | 649 | 1.48 | 1.34 | -45.25 | 350.26 | <.001 | 3.64 |
| Conduct problems | 213 | 1.6 | 1.27 | 649 | 1.21 | 0.81 | -4.25 | 270.35 | <.001 | 0.42 |
| Hyperactivity | 213 | 3.32 | 1.61 | 649 | 2.27 | 1.59 | -8.3 | 356.18 | <.001 | 0.66 |
| Peer problems | 213 | 2.57 | 1.36 | 649 | 1.14 | 0.77 | -14.54 | 257.77 | <.001 | 1.5 |
| Prosocial behaviour | 213 | 8.49 | 1.59 | 649 | 9.11 | 1.03 | 5.35 | 272.45 | <.001 | -0.52 |
| Cluster 3 [social] |  |  |  |  |  |  |  |  |  |  |
| Emotion problems | 210 | 1.98 | 1.52 | 649 | 1.48 | 1.34 | -4.27 | 320.74 | <.001 | 0.36 |
| Conduct problems | 210 | 1.62 | 1.12 | 649 | 1.21 | 0.81 | -4.93 | 282.54 | <.001 | 0.46 |
| Hyperactivity | 210 | 2.63 | 1.6 | 649 | 2.27 | 1.59 | -2.91 | 351.86 | .004 | 0.23 |
| Peer problems | 210 | 3.38 | 1.26 | 649 | 1.14 | 0.77 | -24.35 | 261.38 | <.001 | 2.46 |
| Prosocial behaviour | 210 | 7.91 | 1.75 | 649 | 9.11 | 1.03 | 9.41 | 256.97 | <.001 | -0.97 |

*Note.* Results based on two-tailed Welch *t*-tests. Ext. = externalising; Int. = internalising.

**Table S6**

*Cognitive profiles by cluster at T2*

| Cognitive task | *N* | *M* | *SD* | *N* | *M* | *SD* | *t* | *df* | *p* | *d* |  |
| --- | --- | --- | --- | --- | --- | --- | --- | --- | --- | --- | --- |
| Ext. cluster |  |  |  | Non-elevated difficulties | | |  |  |  |  | |
| SWM Between errors | 220 | 12.26 | 11.5 | 605 | 11.55 | 11.33 | -0.78 | 383.99 | .44 | 0.06 |  |
| CGT Delay aversion | 221 | 0.18 | 0.14 | 608 | 0.16 | 0.13 | -1.66 | 360.23 | .10 | 0.14 |  |
| CGT Risk adjustment | 221 | 1.93 | 1.07 | 612 | 2.04 | 0.95 | 1.35 | 354.91 | .18 | -0.11 |  |
| CGT Risk taking | 221 | 0.59 | 0.13 | 611 | 0.57 | 0.12 | -2.45 | 373.25 | .01 | 0.2 |  |
| Int. cluster |  |  |  |  |  |  |  |  |  |  |  |
| SWM Between errors | 199 | 13.78 | 12.5 | 605 | 11.55 | 11.33 | -2.23 | 312.91 | .03 | 0.19 |  |
| CGT Delay aversion | 198 | 0.16 | 0.15 | 608 | 0.16 | 0.13 | 0.46 | 296.56 | .65 | -0.04 |  |
| CGT Risk adjustment | 199 | 1.94 | 1.04 | 612 | 2.04 | 0.95 | 1.2 | 314.5 | .23 | -0.1 |  |
| CGT Risk taking | 199 | 0.54 | 0.14 | 611 | 0.57 | 0.12 | 2.75 | 311.21 | .01 | -0.24 |  |
| Social cluster |  |  |  |  |  |  |  |  |  |  |  |
| SWM Between errors | 189 | 11.69 | 11 | 605 | 11.55 | 11.33 | -0.15 | 322.42 | .88 | 0.01 |  |
| CGT Delay aversion | 189 | 0.18 | 0.15 | 608 | 0.16 | 0.13 | -1.54 | 286.2 | .12 | 0.14 |  |
| CGT Risk adjustment | 192 | 1.99 | 1.06 | 612 | 2.04 | 0.95 | 0.5 | 294.06 | .62 | -0.04 |  |
| CGT Risk taking | 192 | 0.56 | 0.13 | 611 | 0.57 | 0.12 | 0.31 | 305.45 | .75 | -0.03 |  |

*Note*. Results based on two-tailed Welch *t*-tests. SWM = Spatial working memory; CGT = Cambridge Gambling Task; Ext. = externalising; Int. = internalising.

**Table S7**

*Comparisons of the T1 cognitive function of participants with emerging mental health symptoms at T2 to those with stable non-eleveated difficulties.*

| Transition | *N* | *M* | *SD* | *N* | *M* | *SD* | *t* | *df* | *p* | *d* |
| --- | --- | --- | --- | --- | --- | --- | --- | --- | --- | --- |
| T1 NED to T2 Ext. |  |  |  | NED T1 and T2 | | |  |  |  |  |
| SWM Between errors | 58 | 13.33 | 10.5 | 323 | 17.11 | 13.837 | -2.392 | 96.431 | .019 | 0.283 |
| CGT Delay aversion | 48 | 0.215 | 0.13 | 277 | 0.23 | 0.135 | -0.731 | 64.994 | .467 | 0.113 |
| CGT Risk adjustment | 49 | 1.665 | 0.96 | 279 | 1.718 | 0.996 | -0.354 | 67.538 | .725 | 0.053 |
| CGT Risk taking | 49 | 0.57 | 0.13 | 279 | 0.534 | 0.134 | 1.883 | 68.982 | .064 | -0.278 |
| T1 NED to T2 Int. |  |  |  |  |  |  |  |  |  |  |
| SWM Between errors | 63 | 19.14 | 14.1 | 323 | 17.11 | 13.837 | 1.051 | 86.815 | .296 | -0.147 |
| CGT Delay aversion | 52 | 0.246 | 0.16 | 277 | 0.23 | 0.135 | 0.663 | 64.857 | .510 | -0.114 |
| CGT Risk adjustment | 53 | 1.457 | 0.95 | 279 | 1.718 | 0.996 | -1.815 | 75.204 | .074 | 0.265 |
| CGT Risk taking | 53 | 0.495 | 0.14 | 279 | 0.534 | 0.134 | -1.899 | 72.931 | .062 | 0.286 |
| T1 NED to T2 Social |  |  |  |  |  |  |  |  |  |  |
| SWM Between errors | 61 | 16.62 | 11.9 | 323 | 17.11 | 13.837 | 0.283 | 93.455 | .778 | -0.036 |
| CGT Delay aversion | 58 | 0.235 | 0.16 | 277 | 0.23 | 0.135 | -0.214 | 76.14 | .831 | 0.034 |
| CGT Risk adjustment | 58 | 1.691 | 0.91 | 279 | 1.718 | 0.996 | 0.2 | 88.123 | .842 | -0.027 |
| CGT Risk taking | 58 | 0.538 | 0.12 | 279 | 0.534 | 0.134 | -0.254 | 86.921 | .800 | 0.035 |

*Note*. Results based on two-tailed Welch *t*-tests. SWM = Spatial working memory; CGT = Cambridge Gambling Task; NED = non-elevated difficulties; Ext. = externalising; Int. = internalising.

**Table S8**

*Comparisons of the T1 cognitive function of participants with persisting mental health symptoms at T2 to those with stable non-elevated difficulties.*

| Transition | *N* | *M* | *SD* | *N* | *M* | *SD* | *t* | *df* | *p* | *d* |
| --- | --- | --- | --- | --- | --- | --- | --- | --- | --- | --- |
| Ext. T1 and T2 Ext. |  |  |  | NED T1 and T2 | | |  |  |  |  |
| SWM Between errors | 105 | 21.31 | 14.2 | 323 | 17.11 | 13.837 | 2.647 | 172.69 | .009 | -0.302 |
| CGT Delay aversion | 85 | 0.243 | 0.14 | 277 | 0.23 | 0.135 | 0.722 | 134.09 | .471 | -0.092 |
| CGT Risk adjustment | 85 | 1.417 | 0.91 | 279 | 1.718 | 0.996 | -2.612 | 150.37 | .010 | 0.309 |
| CGT Risk taking | 85 | 0.565 | 0.16 | 279 | 0.534 | 0.134 | 1.647 | 122.64 | .102 | -0.224 |
| Int. T1 and T2 Int. |  |  |  |  |  |  |  |  |  |  |
| SWM Between errors | 59 | 20.14 | 14.8 | 323 | 17.11 | 13.837 | 1.458 | 77.566 | .149 | -0.217 |
| CGT Delay aversion | 50 | 0.237 | 0.14 | 277 | 0.23 | 0.135 | 0.35 | 67.872 | .727 | -0.054 |
| CGT Risk adjustment | 50 | 1.611 | 1.09 | 279 | 1.718 | 0.996 | -0.65 | 64.641 | .518 | 0.106 |
| CGT Risk taking | 50 | 0.497 | 0.14 | 279 | 0.534 | 0.134 | -1.728 | 66.468 | .089 | 0.273 |
| Social T1 and T2 Social |  |  |  |  |  |  |  |  |  |  |
| SWM Between errors | 67 | 19.36 | 14 | 323 | 17.11 | 13.837 | -1.199 | 94.547 | .233 | 0.163 |
| CGT Delay aversion | 64 | 0.263 | 0.13 | 277 | 0.23 | 0.135 | -1.793 | 97.501 | .076 | 0.242 |
| CGT Risk adjustment | 64 | 1.373 | 0.96 | 279 | 1.718 | 0.996 | 2.565 | 96.303 | .012 | -0.349 |
| CGT Risk taking | 64 | 0.534 | 0.15 | 279 | 0.534 | 0.134 | -0.006 | 88.804 | .995 | 0.001 |

*Note*. Results based on two-tailed Welch *t*-tests. SWM = Spatial working memory; CGT = Cambridge Gambling Task; NED = non-elevated difficulties; Ext. = externalising; Int. = internalising.

**Table S9**

*Comparisons of the T1 cognitive function of participants with resolving mental health symptoms at T2 to those with stable non-elevated difficulties.*

| Transition | *N* | *M* | *SD* | *N* | *M* | *SD* | *t* | *df* | *p* | *d* |
| --- | --- | --- | --- | --- | --- | --- | --- | --- | --- | --- |
| T1 Ext. to T2 NED |  |  |  | Ext. T1 and T2 | | |  |  |  |  |
| SWM Between errors | 134 | 18.73 | 13 | 105 | 21.31 | 14.211 | 1.441 | 213.55 | 0.151 | -0.191 |
| CGT Delay aversion | 111 | 0.245 | 0.15 | 85 | 0.243 | 0.142 | -0.116 | 182.63 | 0.908 | 0.017 |
| CGT Risk adjustment | 112 | 1.65 | 1.13 | 85 | 1.417 | 0.91 | -1.606 | 194.19 | 0.110 | 0.226 |
| CGT Risk taking | 112 | 0.558 | 0.14 | 85 | 0.565 | 0.159 | 0.31 | 170.93 | 0.757 | -0.045 |
| T1 Int. to T2 NED |  |  |  | Int. T1 and T2 | | |  |  |  |  |
| SWM Between errors | 71 | 19.45 | 12.9 | 59 | 20.14 | 14.828 | 0.278 | 115.84 | 0.781 | -0.05 |
| CGT Delay aversion | 63 | 0.271 | 0.16 | 50 | 0.237 | 0.135 | -1.229 | 110.28 | 0.222 | 0.231 |
| CGT Risk adjustment | 63 | 1.573 | 0.94 | 50 | 1.611 | 1.085 | 0.195 | 97.365 | 0.846 | -0.038 |
| CGT Risk taking | 63 | 0.509 | 0.13 | 50 | 0.497 | 0.139 | -0.484 | 100.29 | 0.629 | 0.094 |
| T1 Social to T2 NED |  |  |  | Social T1 and T2 | | |  |  |  |  |
| SWM Between errors | 97 | 17.02 | 11.6 | 67 | 19.36 | 14.027 | -1.125 | 123.97 | 0.263 | 0.186 |
| CGT Delay aversion | 85 | 0.225 | 0.14 | 64 | 0.263 | 0.129 | -1.701 | 140.35 | 0.091 | 0.281 |
| CGT Risk adjustment | 85 | 1.667 | 0.93 | 64 | 1.373 | 0.964 | 1.871 | 133.02 | 0.064 | -0.313 |
| CGT Risk taking | 85 | 0.517 | 0.16 | 64 | 0.534 | 0.147 | -0.681 | 141.15 | 0.497 | 0.112 |

*Note*. Results based on two-tailed Welch *t*-tests. SWM = Spatial working memory; CGT = Cambridge Gambling Task; NED = non-elevated difficulties; Ext. = externalising; Int. = internalising.

***Simple transitions***

To explore simple transitions in mental health between mid- to late-adolescence adolescents were classified as having elevated mental health difficulties if they were in any of the three clusters (internalising, externalising or social), and as not having mental health difficulties if they were in the NED group. In other words, at each timepoint, the three “elevated difficulties” clusters were collapsed. This enabled us to explore three simple transitions: i) having mental health symptoms of any type at T1 that resolve by T2 (resolving); ii) having mental health symptoms of any type at T1 that persist in any form at T2, and; iii) having no mental health symptoms at T1, but symptoms of any type at T2 (emerging). We also recorded the number of adolescents with non-elevated difficulties at T1 and T2. The number and percent of adolescents following each of these transitions is shown in Table S10.

**Table S10**

*Simple transitions between mid- (T1) and late- (T2) adolescence*

| Simple Transition | N | % | *p* |
| --- | --- | --- | --- |
| Non-elevated difficulties T1 and T2 | 333 | 26 | .68 |
| Emerging difficulties T1 to T2 | 187 | 14 | <.001 |
| Persisting difficulties T1 to T2 | 468 | 36 | <.001 |
| Resolving difficulties T1 to T2 | 316 | 24 | .54 |

*Note. p*-values denote chi-square results testing the hypothesis that each transition is not statistically different from chance.

The largest proportion of adolescents had persisting difficulties from T1 to T2 (36%), with about a quarter of the sample either having a stable profile of non-elevated difficulties over time or problems that resolved. The emergence of mental health symptoms over time was much rarer, characterising approximately 14% of the sample.

***Simple transitions and cognitive function***

The baseline (T1) cognitive function of the emerging and persisting transitioning groups was compared to that of the group who had non-elevated difficulties across time in a series of planned comparisons. Consistent with the cluster-specific comparisons, the resolving and persisting groups were also compared (see Table 1). The results revealed that those who had persistent mental health problems had poorer spatial working memory and were less able to adjust their risk-related behaviours than those with a stable profile of non-elevated difficulties. There were no other significant group differences (see Figure S2, Table S11).

**Figure S2**

*Differences in T1 cognitive performance for those with resolving, emerging or persisting mental health problems (irrespective of type or profile of mental health symptoms) relative to the NED group.*

*
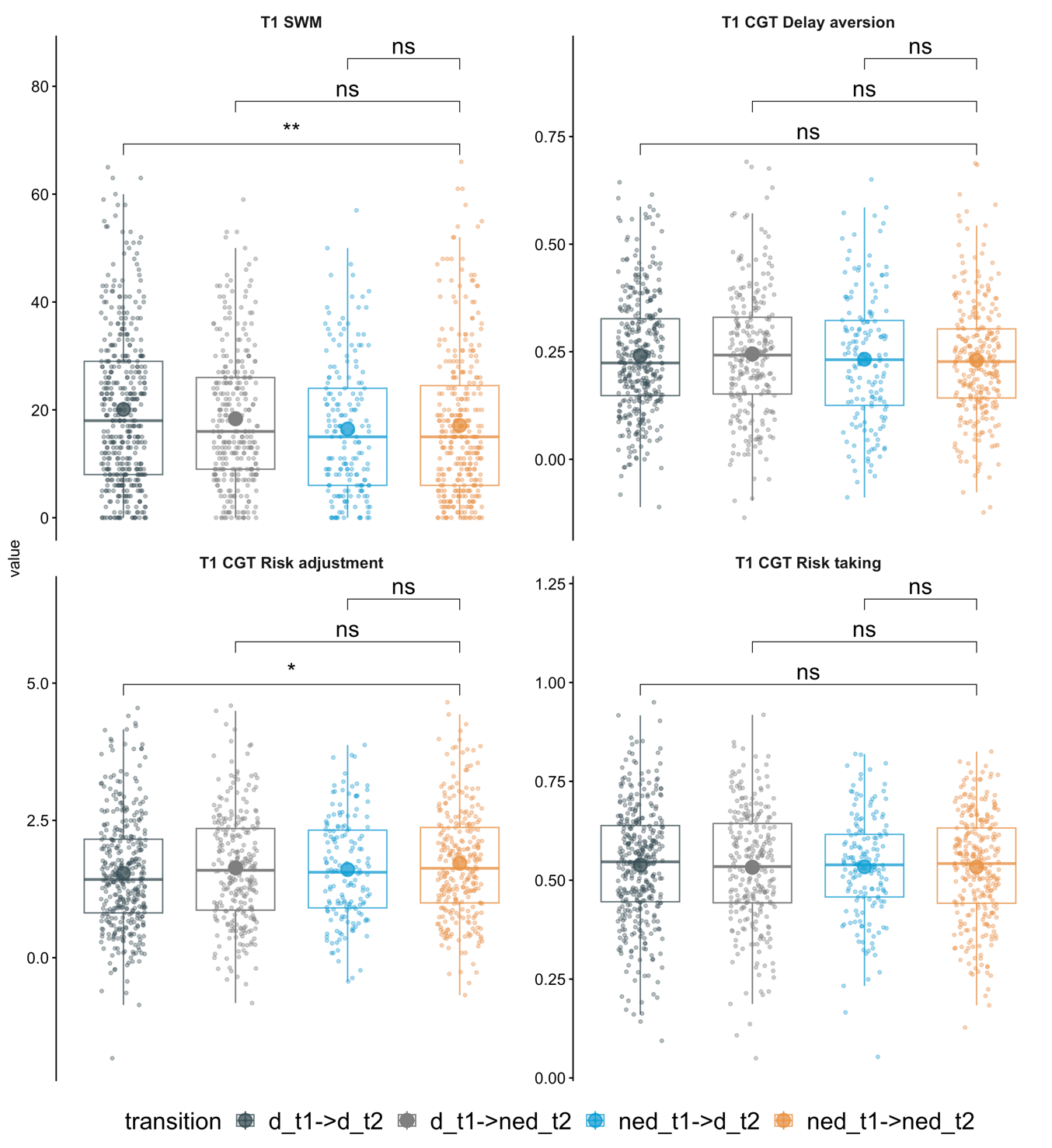
Note.* Results based on two-tailed Welch t-tests. *=p<.05, **=p<.01; ns = non-significant. SWM = Spatial working memory; CGT = Cambridge Gambling Task; d =elevated difficulties (i.e. either Cluster 1,2,or 3); ned= non-elevated difficulties group.

**Table S11**

*Comparisons of the T1 cognitive function of participants with any mental health symptoms at T2 relative to non-elevated difficulties group*

| Transition | *N* | *M* | *SD* | *N* | *M* | *SD* | *t* | *df* | *p* | *d* |
| --- | --- | --- | --- | --- | --- | --- | --- | --- | --- | --- |
| Persisting: ED T1 and T2 |  |  |  | NED T1 and T2 | | |  |  |  |  |
| SWM Between errors | 441 | 20.09 | 14.2 | 323 | 17.11 | 13.837 | 2.913 | 703.21 | .004 | -0.037 |
| CGT Delay aversion | 380 | 0.241 | 0.14 | 277 | 0.23 | 0.135 | 0.977 | 598.16 | .329 | 0.124 |
| CGT Risk adjustment | 380 | 1.536 | 1.02 | 279 | 1.718 | 0.996 | -2.291 | 608.14 | .022 | 0.019 |
| CGT Risk taking | 380 | 0.538 | 0.15 | 279 | 0.534 | 0.134 | 0.447 | 626.2 | .655 | -0.056 |
| Emerging: NED T1 to ED T2 |  |  |  | NED T1 and T2 | | |  |  |  |  |
| SWM Between errors | 182 | 16.45 | 12.5 | 323 | 17.11 | 13.837 | -0.549 | 408.63 | .583 | 0.035 |
| CGT Delay aversion | 158 | 0.232 | 0.15 | 277 | 0.23 | 0.135 | 0.161 | 297.9 | .872 | 0.01 |
| CGT Risk adjustment | 160 | 1.606 | 0.94 | 279 | 1.718 | 0.996 | -1.18 | 347.98 | .239 | 0.047 |
| CGT Risk taking | 160 | 0.534 | 0.13 | 279 | 0.534 | 0.134 | 0.019 | 338.43 | .985 | 0.007 |
| Resolving: ED T1 to NED T2 |  |  |  | Persisting: ED T1 and T2 | | |  |  |  |  |
| SWM Between errors | 302 | 18.35 | 12.5 | 441 | 20.09 | 14.2 | 1.758 | 694.48 | .079 | 0.049 |
| CGT Delay aversion | 259 | 0.245 | 0.15 | 380 | 0.241 | 0.14 | -0.373 | 528.1 | .709 | -0.13 |
| CGT Risk adjustment | 260 | 1.637 | 1.02 | 380 | 1.536 | 1.02 | -1.228 | 558.76 | .22 | -0.054 |
| CGT Risk taking | 260 | 0.533 | 0.15 | 380 | 0.538 | 0.15 | 0.496 | 557.35 | .62 | -0.007 |

*Note*. Results based on two-tailed Welch *t*-tests. SWM = Spatial working memory; CGT = Cambridge Gambling Task; NED = non-elevated difficulties. ED = elevated difficulties (either cluster 1, 2, or 3).

**References**

1. Chaarani B, Spechler PA, Hudson KE, et al (2017) The neural basis of response inhibition and substance abuse. In: Egner T (ed) The Wiley handbook of cognitive control. Wiley Blackwell, pp 581–601

2. Crone EA, Van Duijvenvoorde ACK, Peper JS (2016) Annual Research Review: Neural contributions to risk-taking in adolescence - Developmental changes and individual differences. J Child Psychol Psychiatry 57:353–368. https://doi.org/10.1111/jcpp.12502

3. Nigg JT, Wong MM, Martel MM, et al (2006) Poor response inhibition as a predictor of problem drinking and illicit drug use in adolescents at risk for alcoholism and other substance use disorders. J Am Acad Child Adolesc Psychiatry 45:468–475. https://doi.org/10.1097/01.chi.0000199028.76452.a9

4. Pollak Y, Dekkers TJ, Shoham R, Huizenga HM (2019) Risk-Taking Behavior in Attention Deficit/Hyperactivity Disorder (ADHD): a Review of Potential Underlying Mechanisms and of Interventions. Curr Psychiatry Rep 21:1–11. https://doi.org/10.1007/s11920-019-1019-y

5. Revelle W (2019) psych: Procedures for Psychological, Psychometric, and Personality Research

6. Charrad M, Ghazzali N, Boiteau V, Niknafs A (2014) NbClust: An R Package for Determining the Relevant Number of Clusters in a Data Set. J Stat Softw 61:1–36. https://doi.org/10.18637/JSS.V061.I06

7. Dalmaijer ES, Nord CL, Astle DE (2022) Statistical power for cluster analysis. BMC Bioinformatics 23:1–28. https://doi.org/10.1186/s12859-022-04675-1
